# Comparative neutralization profiles of naive and breakthrough infections with Delta, Omicron BA.1 and BA.2 variants of SARS-CoV-2

**DOI:** 10.1038/s41392-022-01166-w

**Published:** 2022-09-09

**Authors:** Yang Yang, Xiaohua Gong, Jun Wang, Shisong Fang, Jiaqi Zhang, Xuejiao Liao, Yuan Guan, Weihua Wu, Yingxia Liu, Hongzhou Lu

**Affiliations:** 1 Shenzhen Key Laboratory of Pathogen and Immunity, National Clinical Research Center for Infectious Disease, State Key Discipline of Infectious Disease, Shenzhen Third People’s Hospital, Second Hospital Affiliated to Southern University of Science and Technology, Shenzhen, China; 2 Shenzhen Center for Disease Control and Prevention, Shenzhen, China

**Keywords:** Infectious diseases, Infection

**Dear Editor**,

Developing a variant-specific vaccine has drawn great concern due to the considerably altered antigenicity and immune evasion of the Omicron variant. Several recent studies have evaluated the immunogenicity of Omicron-based mRNA vaccines for both regular and booster vaccination and compared it with the wild-type (WT) SARS-CoV-2 counterpart in animal models.^1–3^ However, whether an Omicron-specific mRNA vaccine boost could induce stronger immunity against the Omicron variant remained controversial, which might associate with the differences in the design, modification, and composition of antigens, the experimental settings, and the used animal models. Here we enrolled a total of 215 participants confirmed for naive or breakthrough infections with Delta (*N* = 46), Omicron BA.1 (*N* = 47), and BA.2 variants (*N* = 122) (see detailed information in supplementary Tables S1,S2) and comparatively analyzed the neutralizing profiles against Delta, BA.1 and BA.2 and BA.4/5 variants based on the widely used SARS-CoV-2 pseudovirus neutralization assays.

Participants with naive infections were firstly analyzed. For the Delta naive infections, about 82.35% (14/17) of the samples showed a detectable 50% inhibitory dose (ID_50_) (ID_50_ ≥20) against the Delta variant, while only 35.29% (6/17) for BA.1 and BA.2 variants, and 11.76% (2/17) for BA.4/5 variant. The geometric mean neutralizing titers (GMTs) against Delta, BA.1, BA.2, and BA.4/5 variants were 145.26, 35.34, 22.17, and 15.64, respectively, with reduction folds of 4.11, 6.55, and 9.35 for BA.1, BA.2, and BA.4/5 variants when compared with Delta variant (Fig. 1a). For the BA.1 naive infections, about 66.67% (6/9) of the samples showed detectable ID_50_ against BA.1 and BA.2 variants, while only 11.11% (1/9) for the BA.4/5 variant and 22.22% (2/9) for the Delta variant. The GMTs against BA.1, BA.2, BA.4/5, and Delta variants were 80.30, 27.38, 13.97, and 14.38, respectively, with reduction folds of 2.93 for the BA.2 variant, 5.75 for the BA.4/5 variant and 5.58 for the Delta variants when compared with BA.1 variant (Fig. 1b). As to the BA.2 naive infections, about 67.39% (31/46), 54.35% (25/46), 47.83% (22/46), and 45.65% (21/46) of the samples showed detectable ID_50_ against BA.2, BA.1, BA.4/5 and Delta variants with GMTs of 32.2, 26.18, 23.05, and 19.53, respectively, and the reduction folds were 1.23 for the BA.1 variant, 1.39 for the BA.4/5 variant and 1.65 for the Delta variants when compared with BA.2 variant (Fig. 1c).

**Fig. 1:**
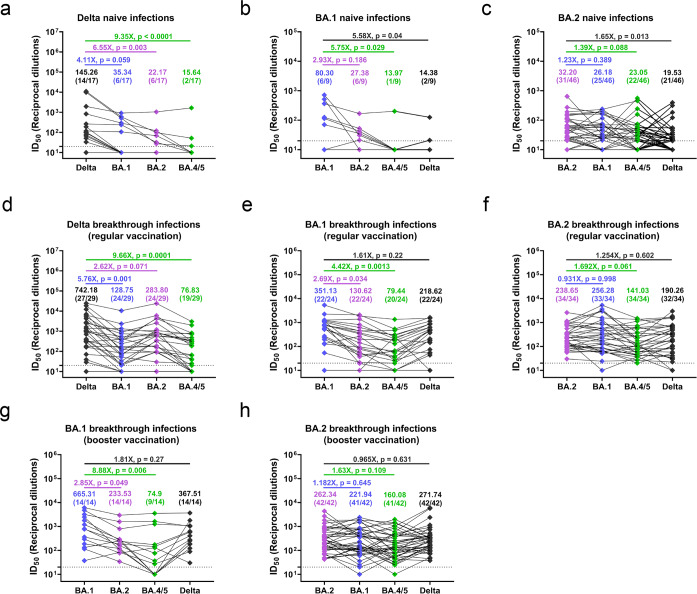
Neutralizing profiles of naive and breakthrough infection with Delta, Omicron BA.1, and BA.2 variants. **a–c** ID_50_ against Delta, BA.1, BA.2, and BA.4/5 variants of samples from naive infections with Delta (**a**), BA.1 (**b**), and BA.2 (**c**) variant. **d–f** ID_50_ against Delta, BA.1, BA.2, and BA.4/5 variants of samples from breakthrough infections with Delta (**d**), BA.1 (**e**), and BA.2 (**f**) variant in regular vaccinees. **g**, **h** ID_50_ against Delta, BA.1, BA.2, and BA.4/5 variants of samples from breakthrough infections with BA.1 (**g**) and BA.2 (**h**) variant in booster vaccinees. For all panels, values above the symbols denote geometric mean titers, and the numbers in parentheses denote the proportion of positive plasma with ID_50_ above the limit of detection (dotted lines, ≥1:20). Values above the transverse lines denote fold changes and *p* values. Statistical significance was determined using Mann–Whitney *U*-test, and *p* values less than 0.05 were considered statistically significant

Then we analyzed participants with breakthrough infections. In the Delta group with regular vaccination (*N* = 29; 28 participants with two doses of inactivated vaccine and one participant with two doses of mRNA vaccine), about 93.1% (27/29), 82.76% (24/29), 82.76% (24/29), and 65.52% (19/29) of the samples showed detectable ID_50_ against Delta, BA.1, BA.2, and BA.4/5 variants with GMTs of 742.18, 128.75, 283.80, and 76.83, respectively, and the reduction folds were 5.76 for the BA.1 variant, 2.62 for the BA.2 variant, and 9.66 for the BA.4/5 variant when compared with Delta variant (Fig. 1d). For the BA.1 group with regular vaccination (*N* = 24; 14 participants received two doses of inactivated vaccine and 10 participants two doses of mRNA vaccine), about 91.67% (22/24) of the samples showed detectable ID_50_ against BA.1, BA.2 and Delta variants, and 83.33% (20/24) against BA.4/5 variant with GMTs of 351.13, 130.62, 218.62, and 79.44, respectively. The reduction folds were 2.69 for the BA.2 variant, 4.42 for the BA.4/5 variant, and 1.61 for the Delta variant when compared with BA.1 variant (Fig. 1e). For the BA.2 group with regular vaccination (*N* = 34; 29 participants with two doses of inactivated vaccine, three participants with two doses of mRNA vaccine and two participants with three doses of protein subunit vaccine), all the samples (34/34) showed detectable ID_50_ against BA.2, BA.4/5 variants, while 97.06 (33/34) for the BA.1 and 94.12% (32/34) for the Delta variant with GMTs of 238.65, 141.03, 256.28, and 190.26, respectively, and the reduction folds were 0.931 for the BA.1 variant, 1.692 for the BA.4/5 variant and 1.254 for the Delta variant when compared with BA.2 variant (Fig. 1f).

For the BA.1 group with booster vaccination (*N* = 14; eight participants with a homologous booster of inactivated vaccine, one participant with a homologous booster of mRNA vaccine, and five participants with a heterogenous booster of mRNA vaccine), all the samples (14/14) showed detectable ID_50_ against BA.1, BA.2, and Delta variants, while only 64.29% (9/14) showed detectable ID_50_ against BA.4/5 variant with GMTs of 665.31, 233.53, 367.51, and 74.9, respectively. The reduction folds were 2.85 for the BA.2 variant, 8.88 for the BA.4/5 variant, and 1.81 for the Delta variant when compared with BA.1 variant (Fig. 1g). As to the BA.2 group with booster vaccination (*N* = 42; 38 participants with a homologous booster of inactivated vaccine, three participants with a homologous booster of mRNA vaccine, and one participant with a heterogenous booster of mRNA vaccine), all the samples (42/42) showed detectable ID_50_ against BA.2 and Delta variants, while 97.62% (41/42) for the BA.1 and BA.4/5 variants with GMTs of 262.34, 271.74, 221.94, and 160.08, respectively. The reduction folds were 1.182 for the BA.1 variant, 1.63 for the BA.4/5 variant, and 0.965 for the Delta variant when compared with BA.2 variant (Fig. 1h). No significant correlation was found between the fold changes and the age (supplementary Fig. S1), the lowest Ct values during hospitalization (supplementary Fig. S4) and the days between last vaccination and laboratory confirmation (supplementary Fig. S5). Moreover, similar fold changes were also found between male and female participants (supplementary Fig. S2) and among participants with different disease severity (supplementary Fig. S3).

Our results for the naive and breakthrough infections with Delta and BA.1 variants showed that limited cross-neutralizing responses were induced, especially for the currently dominant BA.4/5 variant. This is consistent with previous findings that vaccination with BA.1 specific mRNA vaccine alone or infection with BA.1 provided poor cross-protection,^1,4^ and that BA.4/5 variant could significantly escape the immune response induced by BA.1 breakthrough infection.^5^ These observations might result from that BA.1 breakthrough infection predominantly recalls humoral immune memory against the WT SARS-CoV-2 spike protein and the resulting elicited neutralizing antibodies are enriched on epitopes of the spike protein that do not bind ACE2.^6^ As to the BA.2 variant, it was between the BA.1 and BA.4/5 variants phylogenetically, sharing some mutations with either BA.1 or BA.4/5.^7^ Results in our study indicated that it could not confer strong immunity in unvaccinated participants, while a significantly enhanced immune response was found in the breakthrough infections (GMTs: 238.65 and 262.34 vs 32.2). Noteworthy, both naive and breakthrough infections with BA.2 maintained comparable neutralizing activities against other variants, including BA.4/5 variant, indicating a possibly broader cross-immunity induced by BA.2 variant. Waning immunity and immune evasion against variants of concern were common in vaccinees with current COVID-19 vaccines, and variant-matched boosts have been suggested as a strategy to get an enhanced immune response to the corresponding variants beyond boosts with existing vaccines.^3^ However, due to the rapid transmission and evolution associated uncertainty of potential new variants, broad-spectrum protection may be preferable against variant-specific protection in the development of next-generation vaccines. Taking together, Omicron BA.2-based vaccine might be a better candidate than BA.1 for the update of the current vaccines to induce broad-spectrum protection. It should also be noted that the absolute values of cross-neutralizing titers induced by BA.2 were not all distinctly higher than those induced by BA.1 or Delta variant, which indicated a possibly lower immunogenicity of BA.2 variant. Accordingly, enhancement of the immunogenicity of BA.2-based antigen by further optimizing the sequences, the adjuvants, and the immunization schedule was necessary.

There are several limitations of our study. Firstly, we did not compare the differences among different types of vaccines due to limited accessibility to some types of vaccines like mRNA, protein subunit, and adenovirus-based vaccines. Secondly, samples from BA.4/5 infections were not included in the analyses due to inaccessibility. Thirdly, only the pseudotyped virus system was used to evaluate the neutralizing antibody responses. Nevertheless, our results provide important information for the update of the current vaccines.

## Supplementary information

Supplementary Materials

## Data Availability

The data supporting the findings of this study are available within the paper and the supplementary information.
