# Supplementary material for: Comparative neutralization profiles of naive and breakthrough infections with Delta, Omicron BA.1 and BA.2 variants of SARS-CoV-2
Source: Signal Transduct Target Ther. 2022 Sep 9;7:316. doi: 10.1038/s41392-022-01166-w (PMC9462069; doi:10.1038/s41392-022-01166-w)
Supplement: Supplementary file 1 — Supplementary Materials [file 41392_2022_1166_MOESM1_ESM.docx]

Supplementary Materials for

**Comparative neutralization profiles of naive and breakthrough infections with Delta, Omicron BA.1 and BA.2 variants of SARS-CoV-2**

Yang Yang^1^†*, Xiaohua Gong^1^†, Jun Wang^1^†, Shisong Fang^2^, Jiaqi Zhang^1^, Xuejiao Liao^1^, Yuan Guan^1^, Weihua Wu^2^, Yingxia liu^1^*, Hongzhou Lu^1^*

^1^Shenzhen Key Laboratory of Pathogen and Immunity, National Clinical Research Center for Infectious Disease, State Key Discipline of Infectious Disease, Shenzhen Third People's Hospital, Second Hospital Affiliated to Southern University of Science and Technology, Shenzhen, China

^2^Shenzhen Center for Disease Control and Prevention, Shenzhen, China

†Contributed equally.

*Contributed equally.

Correspondence to: Yang Yang, [yyszth2018@163.com;](mailto:yyszth2018@163.com;) Hongzhou Lu, Email: [luhongzhou@szsy.sustech.edu.cn](mailto:luhongzhou@szsy.sustech.edu.cn,); Yingxia Liu, Email: [yingxialiu@hotmail.com](mailto:yingxialiu@hotmail.com);

Materials and Methods

**Patient information and sample collection**

Subjects presented in this study were laboratory confirmed COVID-19 patients using quantitative real-time PCR ((BioGerm, Shanghai, China) (N=215). These participants were further divided into Delta, BA.1 and BA.2 groups based on the genotypes of infecting SARS-CoV-2. The genotype of infected virus in the Delta and BA.1 groups were confirmed by qRT-PCR or sequencing. A total of 87 participants in the BA.2 group were confirmed by sequencing, while the rest were determined according to the date of admission and the epidemiology. Blood samples were collected from the enrolled patients during hospitalization post 7 days post illness onset/laboratory confirmation (d.a.o) and subjected for the tests. Disease severity classification was evaluated according to China National Health Commission Guidelines for Diagnosis and Treatment of SARS-CoV-2 infection (seventh version).

**Viral entry inhibition assay with pseudotyped SARS-CoV-2 virus**

SARS-CoV-2 pseudotype neutralization assays were conducted to test the neutralizing activity of the plasma samples. In brief, plasma samples were heat-inactivated at 56 °C for 30 min to remove complement activity, then serially diluted in 5-folds with DMEM supplemented with 10% FBS from 1:20 to 1:62500 in white, flat-bottom 96-well plates (Thermo Fisher) in a total volume of 50μl. Then 200 TCID_50_ of SARS-CoV-2 pseudotyped particles (Vazyme) in 50 μl were added to each well and incubated at 37°C for 1h. A total of 2 × 10^4^ ACE2-293T cells in 100μl complete media were added per well and incubated for 48h at 37°C and 5% CO_2_. Firefly luciferase activity (luminescence) was measured using Bright-Light^TM^ Luciferase Assay System (Vazyme) and a VARIOSKAN LUX Multi-Mode plate reader (Thermo) according to the manufacture’s protocols. Neutralizing antibody titers were calculated as a 50% inhibitory dose (ID_50_) expressed as the dilution of plasma that resulted in a 50% reduction of luciferase luminescence compared with virus control in single-round pseudovirus infection assay.

**Statistical analysis**

The neutralizing titers of ID_50_ were calculated using a five-parameter dose-response curve in Graphpad Prism. Mann-Whitney U test was used to compare the ID_50_ between two groups. All statistical tests performed using Graphpad Prism. P values less than 0.05 were considered statistically significant.

Supplementary Text

**Supplementary figures.**


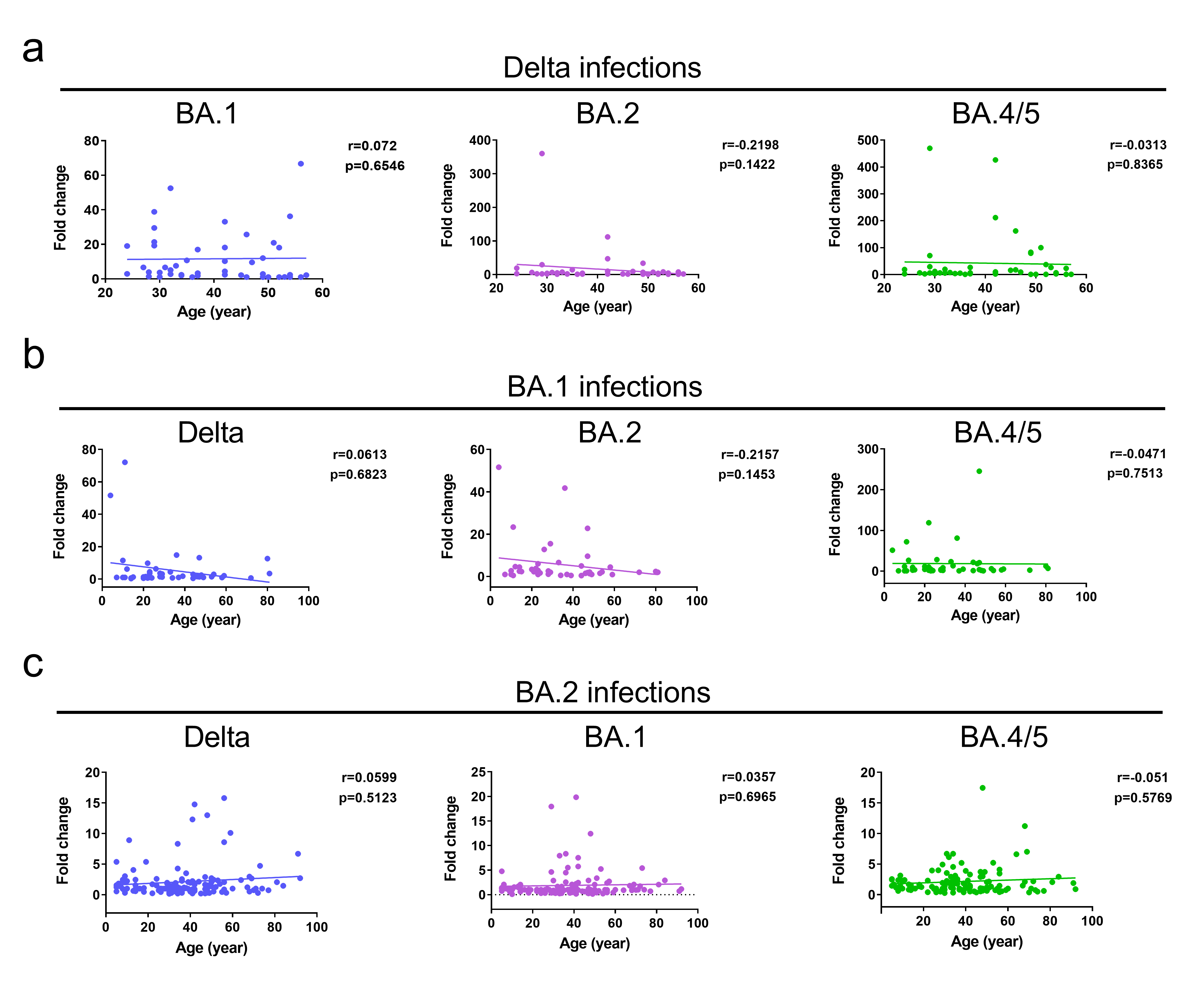


Figure. S1.

**Spearman correlation plot of fold changes of the ID_50_ against the causative agents with age in Delta infections (a), BA.1 infections (b) and BA.2 infections (c).**


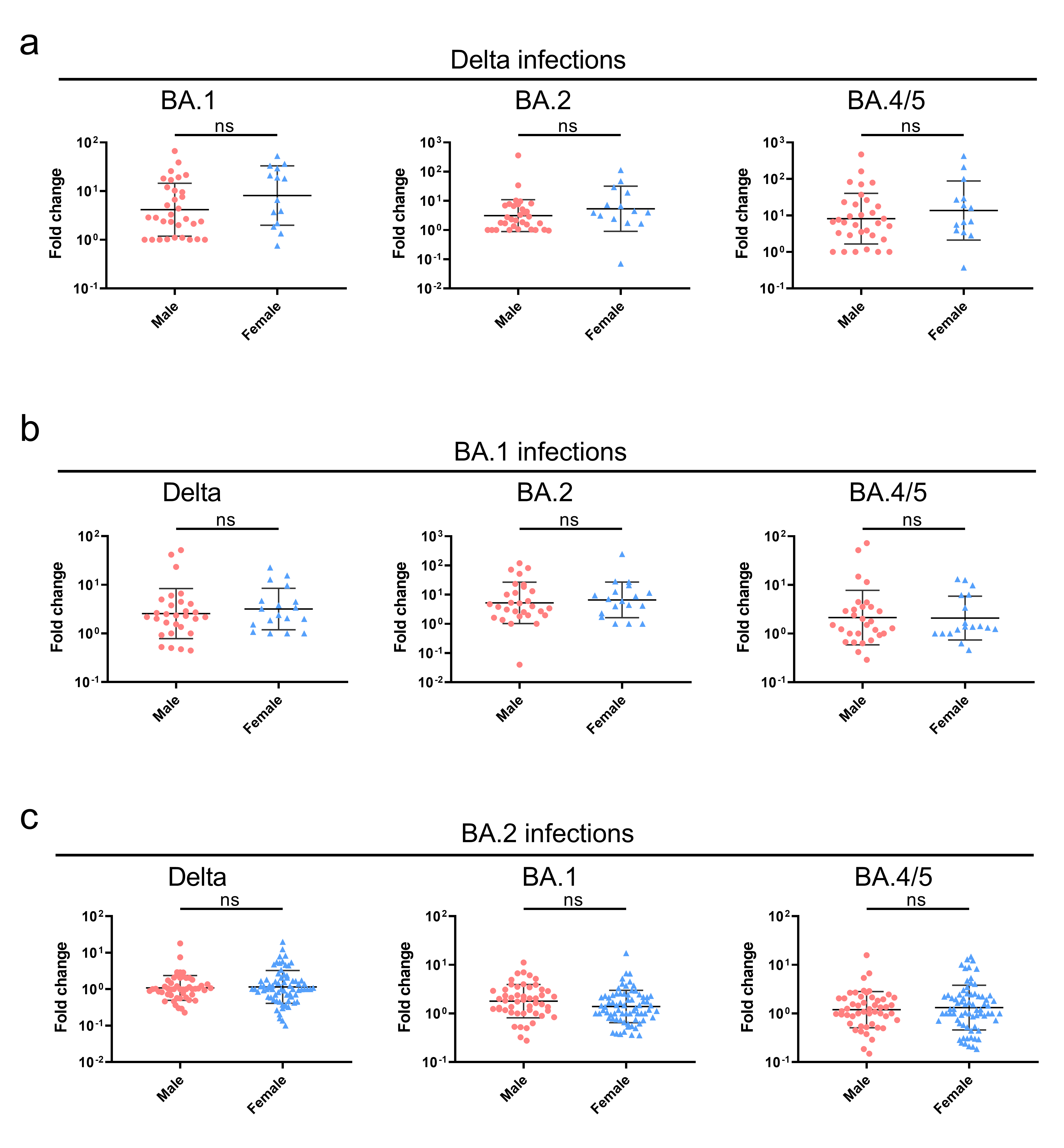


Figure. S2.

**Comparison of fold changes of the ID_50_ against the causative agents between male and female participants with Delta infections (a), BA.1 infections (b) and BA.2 infections (c).**


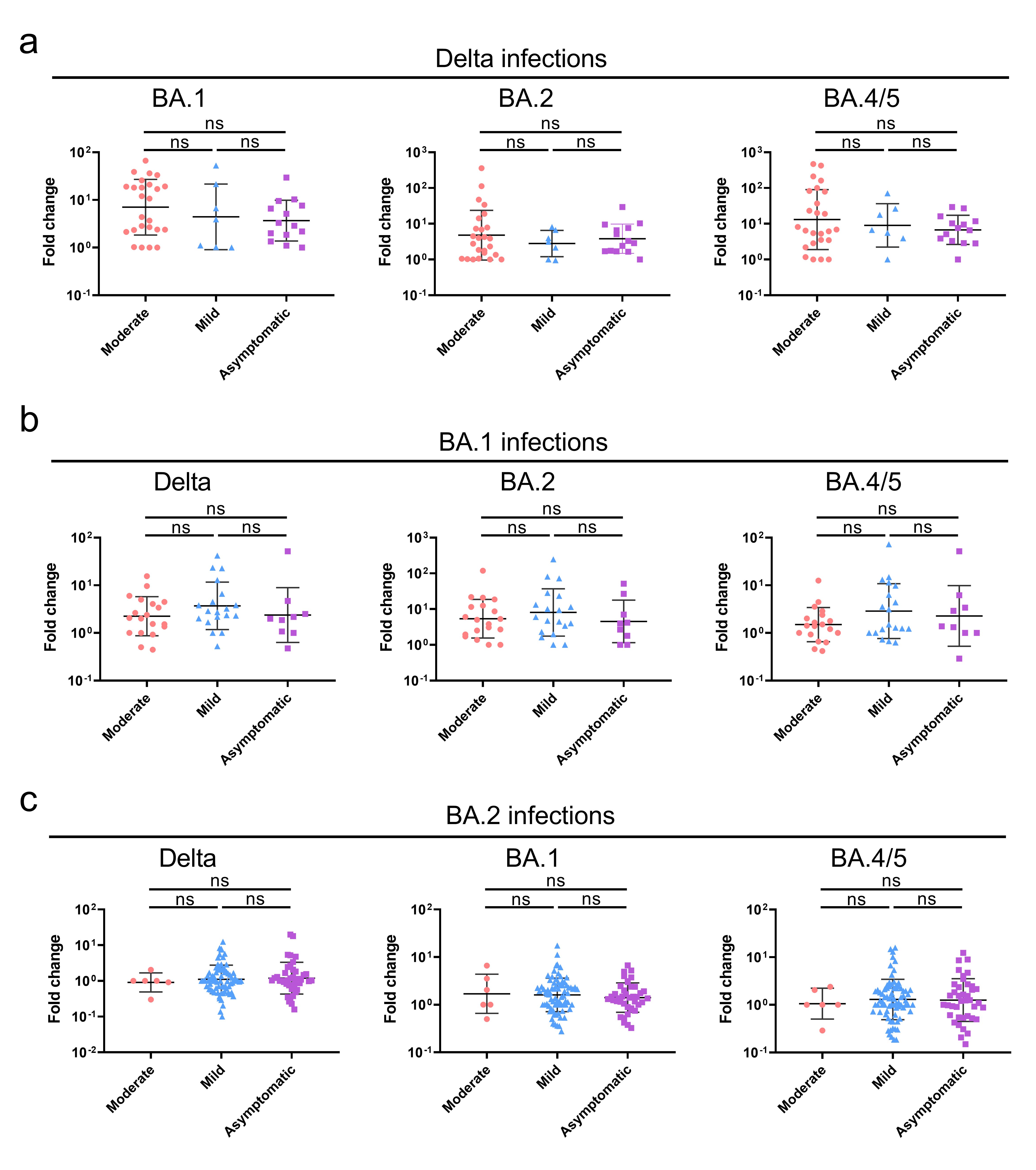


Figure. S3.

**Comparison of fold changes of the ID_50_ against the causative agents among participants with different disease severity in Delta infections (a), BA.1 infections (b) and BA.2 infections (c).**


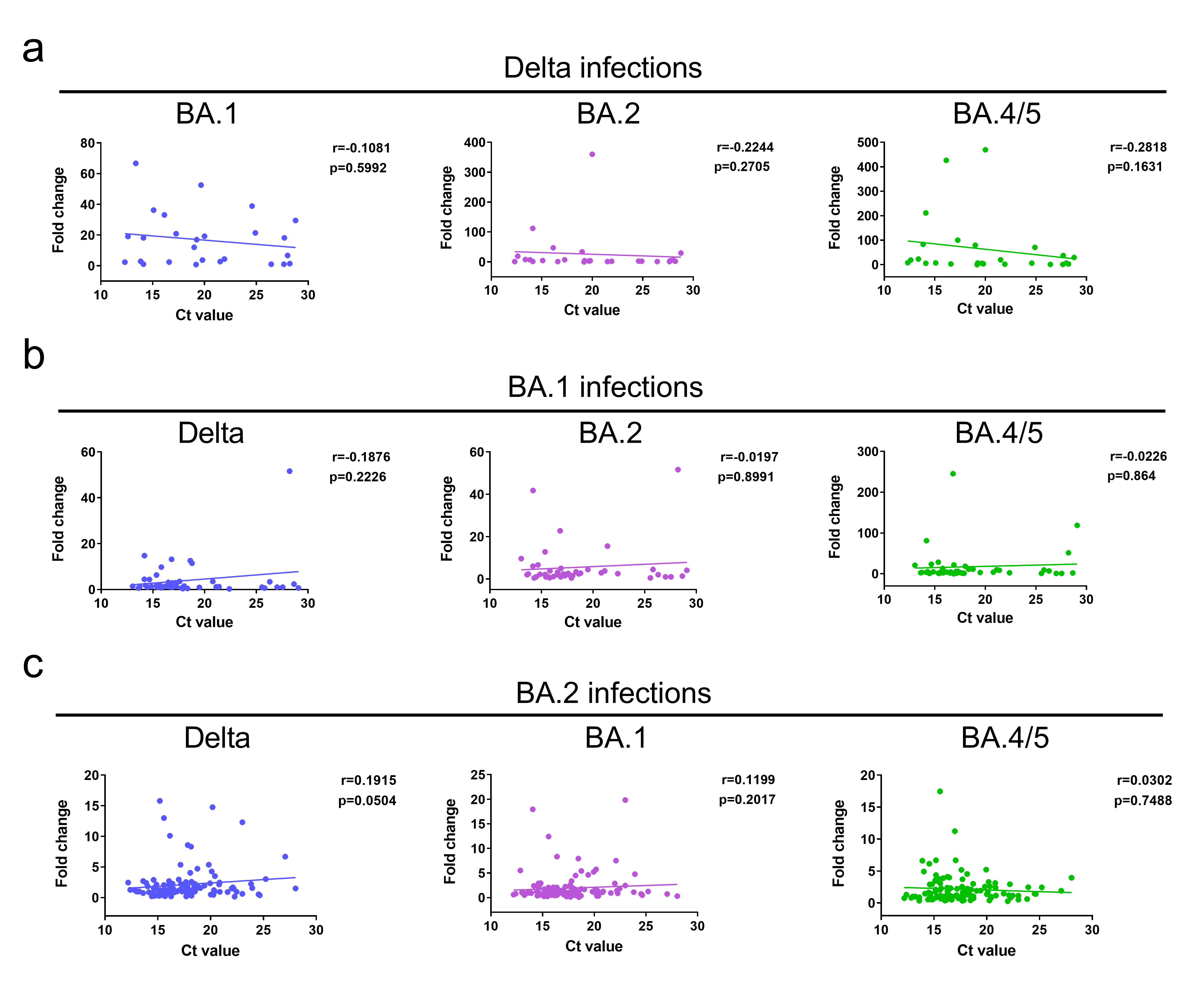


Figure. S4.

**Spearman correlation plot of fold changes of the ID_50_ against the causative agents with the lowest Ct values during hospitalization in Delta infections (a), BA.1 infections (b) and BA.2 infections (c).** Only the samples with the lowest Ct values during hospitalization under 30 were included for the analysis.. Create a page break and paste in the Figure above the caption.


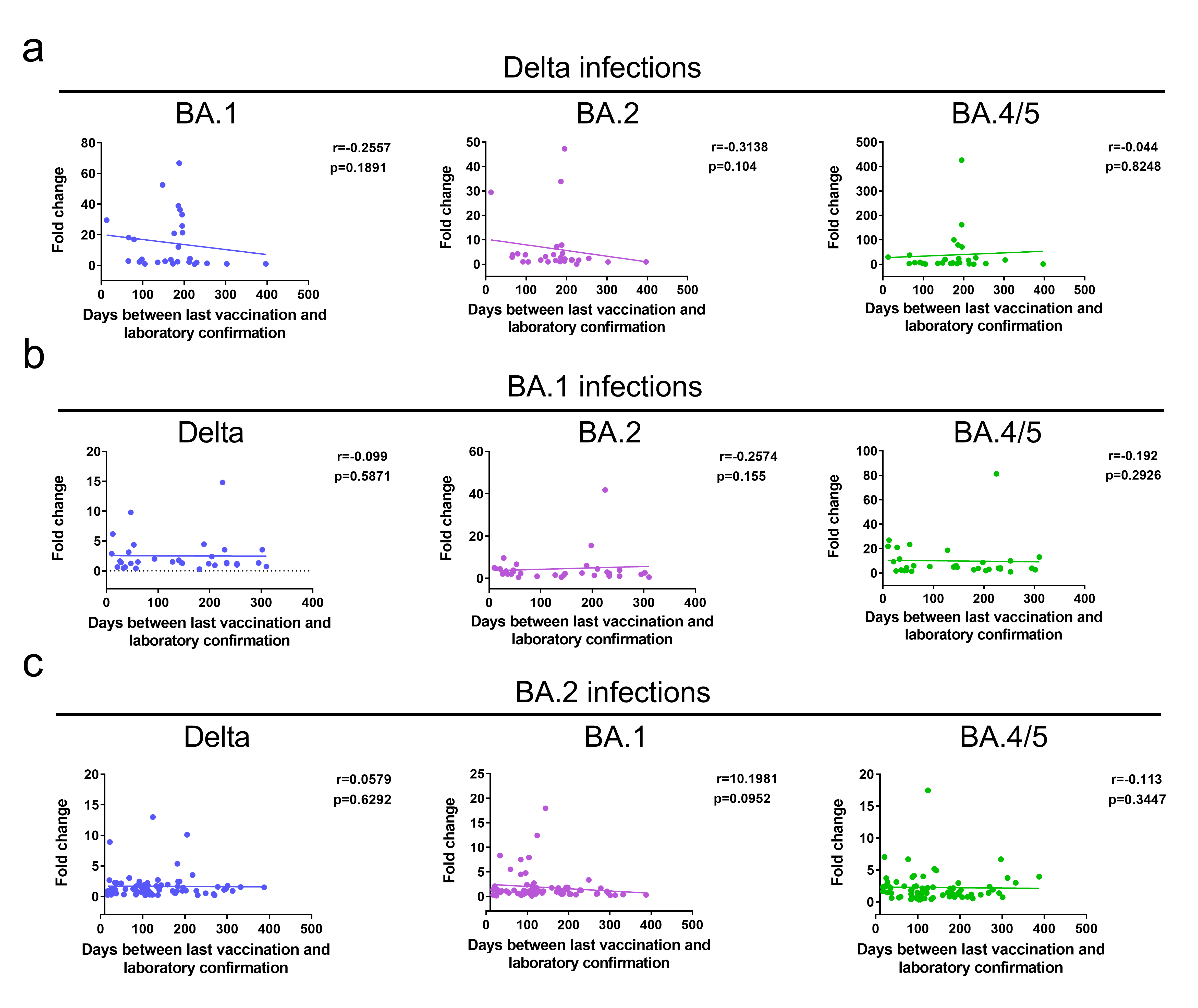


Figure. S5.

**Spearman correlation plot of fold changes compared with the causative agents with the days between last vaccination and laboratory confirmation in Delta infections (a), BA.1 infections (b) and BA.2 infections (c).**

**Supplementary Tables.**

Table S1.

**Summary of the characteristics on subjects included in this study.**

| **Characteristics** | **Delta (N=46)** | | | **Omicron BA.1 (N=47)** | | | **Omicron BA.2 (N=122)** | | |
| --- | --- | --- | --- | --- | --- | --- | --- | --- | --- |
|  | **Unvaccinated (17)** | **Regular vaccination (29)** | **Booster vaccination (0)** | **Unvaccinated (9)** | **Regular vaccination (24)** | **Booster vaccination (14)** | **Unvaccinated (46)** | **Regular vaccination (34)** | **Booster vaccination (42)** |
| **Median age (range)** | 37 (24-57) | 42 (24-56) | NA | 11 (4-81) | 28 (12-59) | 34 (20-72) | 40 (5-92) | 16 (5-77) | 39.5 (5-72) |
| **Male (%, n/N)** | 82.4 (14/17) | 62.1 (18/29) | NA | 55.6 (5/9) | 62.5 (15/24) | 64.3 (9/14) | 52.1 (24/46) | 61.8 (21/34) | 64.3 (27/42) |
| **Age subgroup (%, n/N)** |  |  |  |  |  |  |  |  |  |
| <18 yr | 0 (0/17) | 0 (0/29) | NA | 66.7 (6/9) | 16.7 (4/24) | 0 (0/14) | 10.8 (5/46) | 50 (17/34) | 2.4 (1/42) |
| 18-64 yr | 100 (17/17) | 100 (29/29) | NA | 11.1 (1/9) | 83.3 (20/24) | 92.3 (13 /14) | 76.7 (33/46) | 35.3 (12/34) | 92.9 (39/42) |
| ≥65 yr | 0 (0/17) | 0 (0/29) | NA | 22.2 (2/9) | 0 (0/24) | 7.2 (1/14) | 18.6 (8/46) | 14.7 (5/34) | 4.8 (2/42) |
| **Co-existing chronic medical conditions (%, n/N)** | 5.8 (1/17) | 3.4 (1/29) | NA | 22 (2/9) | 8.3 (2/24) | 14.3 (2/14) | 41.3 (19/46) | 5.9 (2/34) | 7.1 (3/42) |
| **Disease severity (%, n/N)** |  |  |  |  |  |  |  |  |  |
| Asymptomatic | 41.2 (7/17) | 24.1 (7/29) | NA | 55.5 (5/9) | 16.7 (4/24) | 0 (0/14) | 26.1 (12/46) | 44.1 (15/34) | 31.0 (13/42) |
| Mild | 17.6 (3/17) | 13.8 (4/29) | NA | 33.3 (3/9) | 41.7 (10/24) | 42.9 (6/14) | 60.9 (28/46) | 55.9 (19/34) | 69.0 (29/42) |
| Moderate | 41.2 (7/17) | 62.1 (18/29) | NA | 11.1 (1/9) | 41.7 (10/24) | 57.1 (8/14) | 13.0 (6/46) | 0 (0/34) | 0 (0/42) |
| Severe | 0 (0/17) | 0 (0/29) | NA | 0 (0/9) | 0 (0/24) | 0 (0/14) | 0 (0/46) | 0 (0/34) | 0 (0/42) |
| **Median day between vaccination and laboratory confirmation (range)** | NA | 185 (13-397) | NA | NA | 204 (12-302) | 47 (10-310) | NA | 159.5 (21-388) | 105 (17-249) |
| **Median day between illness onset/laboratory confirmation and sample collection (range)** | 21 (7-127) | 10 (7-56) | NA | 13 (7-27) | 8.5 (7-27) | 16 (7-41) | 14 (7-77) | 37 (7-91) | 12.5 (7-91) |

NA: Not applicable.

Table S2.

**Detailed characteristics on subjects included in this study at individual level.**

| **Infected variants** | **Sex** | **Age** | **Co-existing chronic medical conditions** | **Disease severity** | **Groups of vaccination** | **Types of vaccination** | | | **Days between last vaccination and illness onset/laboratory confirmation** | **Days between illness onset/laboratory confirmation and sample collection** | **Lowest Ct values during hospitalization** | **ID_50_** | | | |
| --- | --- | --- | --- | --- | --- | --- | --- | --- | --- | --- | --- | --- | --- | --- | --- |
|  |  |  |  |  |  | **First dose** | **Second dose** | **Third dose** |  |  |  | **Delta** | **BA.1** | **BA.2** | **BA.4/5** |
| Delta | Male | 49 | No | Moderate | Unvaccinated | / | / | / | / | 18 | 13.85 | 831.76 | 293.09 | 120.23 | 10.00 |
| Delta | Male | 27 | No | Mild | Unvaccinated | / | / | / | / | 11 | 38.02 | 66.60 | 10.00 | 10.00 | 10.00 |
| Delta | Female | 31 | No | Asymptomatic | Unvaccinated | / | / | / | / | 10 | 37.98 | 66.06 | 10.00 | 10.00 | 10.00 |
| Delta | Male | 33 | No | Asymptomatic | Unvaccinated | / | / | / | / | 7 | 37.53 | 75.86 | 10.00 | 10.00 | 10.00 |
| Delta | Male | 47 | No | Asymptomatic | Unvaccinated | / | / | / | / | 10 | 37.82 | 95.49 | 10.00 | 10.00 | 10.00 |
| Delta | Male | 42 | No | Asymptomatic | Unvaccinated | / | / | / | / | 7 | 33.21 | 102.33 | 10.00 | 10.00 | 10.00 |
| Delta | Male | 57 | Yes | Moderate | Unvaccinated | / | / | / | / | 8 | 33.02 | 1949.84 | 912.01 | 1047.13 | 1659.60 |
| Delta | Male | 37 | No | Asymptomatic | Unvaccinated | / | / | / | / | 8 | 36.07 | 33.11 | 10.00 | 10.00 | 10.00 |
| Delta | Male | 32 | No | Asymptomatic | Unvaccinated | / | / | / | / | 10 | 33.91 | 51.18 | 10.00 | 10.00 | 10.00 |
| Delta | Male | 56 | No | Mild | Unvaccinated | / | / | / | / | 56 | 34.38 | 10.00 | 10.00 | 10.00 | 10.00 |
| Delta | Male | 30 | No | Asymptomatic | Unvaccinated | / | / | / | / | 8 | 34.14 | 117.49 | 106.65 | 69.18 | 10.00 |
| Delta | Male | 53 | No | Mild | Unvaccinated | / | / | / | / | 41 | 33.02 | 263.03 | 238.00 | 32.36 | 10.00 |
| Delta | Male | 36 | No | Moderate | Unvaccinated | / | / | / | / | 8 | 37.66 | 10.00 | 10.00 | 10.00 | 10.00 |
| Delta | Male | 29 | No | Moderate | Unvaccinated | / | / | / | / | 8 | 20.01 | 9862.79 | 512.86 | 27.38 | 21.00 |
| Delta | Female | 24 | No | Moderate | Unvaccinated | / | / | / | / | 21 | 12.63 | 190.54 | 10.00 | 10.00 | 10.00 |
| Delta | Male | 50 | No | Moderate | Unvaccinated | / | / | / | / | 10 | 33.76 | 10.00 | 10.00 | 10.00 | 10.00 |
| Delta | Female | 42 | No | Moderate | Unvaccinated | / | / | / | / | 20 | 14.12 | 10964.78 | 602.56 | 97.72 | 51.80 |
| Delta | Male | 29 | No | Mild | Regular | Inactivated | Inactivated | / | 196 | 9 | 24.91 | 707.95 | 33.11 | 331.13 | 10.00 |
| Delta | Male | 29 | No | Moderate | Regular | Inactivated | Inactivated | / | 186 | 10 | 24.59 | 12302.68 | 316.83 | 4466.83 | 1995.30 |
| Delta | Male | 49 | No | Moderate | Regular | Inactivated | Inactivated | / | 186 | 10 | 19.02 | 5248.07 | 436.52 | 154.88 | 66.10 |
| Delta | Male | 54 | Yes | Moderate | Regular | Inactivated | Inactivated | / | 184 | 30 | 16.59 | 924.69 | 388.15 | 870.96 | 324.30 |
| Delta | Female | 35 | No | Moderate | Regular | Inactivated | Inactivated | / | 225 | 21 | 19.2 | 301.99 | 398.11 | 4265.79 | 812.80 |
| Delta | Male | 54 | No | Moderate | Regular | Inactivated | Inactivated | / | 173 | 23 | 14.12 | 1380.38 | 1345.86 | 891.25 | 251.20 |
| Delta | Female | 54 | No | Moderate | Regular | Inactivated | Inactivated | / | 190 | 40 | 15.09 | 2630.27 | 72.61 | 588.84 | 380.20 |
| Delta | Male | 56 | No | Moderate | Regular | Inactivated | Inactivated | / | 188 | 27 | 13.38 | 6982.32 | 104.71 | 891.25 | 302.00 |
| Delta | Male | 42 | No | Moderate | Regular | Inactivated | Inactivated | / | 214 | 37 | 21.93 | 1047.12 | 239.88 | 588.84 | 477.50 |
| Delta | Male | 42 | No | Moderate | Regular | Inactivated | Inactivated | / | 92 | 21 | 12.33 | 24547.09 | 10519.62 | 23988.32 | 3019.90 |
| Delta | Male | 46 | No | Moderate | Regular | Inactivated | Inactivated | / | 195 | 9 | 37.83 | 1621.81 | 63.09 | 1202.26 | 10.00 |
| Delta | Female | 37 | No | Asymptomatic | Regular | Inactivated | Inactivated | / | 230 | 8 | 37.56 | 1023.00 | 549.54 | 616.59 | 38.00 |
| Delta | Female | 29 | No | Asymptomatic | Regular | Inactivated | Inactivated | / | 13 | 23 | 36.78 | 295.12 | 10.00 | 10.00 | 10.00 |
| Delta | Male | 32 | No | Moderate | Regular | Inactivated | Inactivated | / | 154 | 27 | 21.52 | 6309.57 | 2344.23 | 6025.59 | 316.20 |
| Delta | Female | 32 | No | Mild | Regular | Inactivated | Inactivated | / | 148 | 23 | 19.67 | 1109.17 | 21.13 | 354.81 | 199.50 |
| Delta | Male | 37 | No | Moderate | Regular | Inactivated | Inactivated | / | 79 | 22 | 19.25 | 407.38 | 23.99 | 95.50 | 63.10 |
| Delta | Female | 51 | No | Moderate | Regular | Inactivated | Inactivated | / | 176 | 31 | 17.27 | 18620.87 | 891.25 | 2570.39 | 186.20 |
| Delta | Female | 30 | No | Moderate | Regular | Inactivated | Inactivated | / | 168 | 23 | 19.81 | 2754.23 | 749.89 | 691.83 | 794.30 |
| Delta | Female | 28 | No | Asymptomatic | Regular | Inactivated | Inactivated | / | 255 | 9 | 38.23 | 70.79 | 52.48 | 29.51 | 25.10 |
| Delta | Female | 45 | No | Asymptomatic | Regular | Inactivated | Inactivated | / | 212 | 54 | 34.53 | 323.59 | 147.91 | 184.50 | 20.00 |
| Delta | Male | 24 | No | Asymptomatic | Regular | mRNA | mRNA | / | 65 | 8 | 36.84 | 28.84 | 10.00 | 10.00 | 10.00 |
| Delta | Male | 34 | No | Asymptomatic | Regular | Inactivated | Inactivated | / | 136 | 7 | 34.74 | 162.18 | 81.28 | 95.50 | 41.70 |
| Delta | Male | 34 | No | Moderate | Regular | Inactivated | Inactivated | / | NA | 9 | 35.76 | 2511.88 | 1047.13 | 1086.43 | 707.95 |
| Delta | Female | 28 | No | Mild | Regular | Inactivated | Inactivated | / | 98 | 7 | 37.23 | 38.91 | 10.00 | 10.00 | 10.00 |
| Delta | Male | 49 | No | Asymptomatic | Regular | Inactivated | Inactivated | / | 397 | 127 | 30.35 | 10.00 | 10.00 | 10.00 | 10.00 |
| Delta | Male | 52 | No | Moderate | Regular | Inactivated | Inactivated | / | 105 | 7 | 36.44 | 10.00 | 10.00 | 10.00 | 10.00 |
| Delta | Female | 42 | No | Moderate | Regular | Inactivated | Inactivated | / | 195 | 12 | 16.14 | 4265.79 | 128.82 | 90.28 | 10.00 |
| Delta | Male | 52 | No | Moderate | Regular | Inactivated | Inactivated | / | 66 | 8 | 37.71 | 371.53 | 20.49 | 95.50 | 10.00 |
| Delta | Male | 46 | No | Mild | Regular | Inactivated | Inactivated | / | 303 | 7 | 32.89 | 177.82 | 173.78 | 186.21 | 10.00 |
| BA.1 | Female | 81 | Yes | Asymptomatic | Unvaccinated | / | / | / | / | 11 | 36.31 | 20.89 | 70.79 | 35.48 | 10.00 |
| BA.1 | Female | 80 | No | Moderate | Unvaccinated | / | / | / | / | 13 | 18.62 | 10.00 | 125.89 | 52.48 | 10.00 |
| BA.1 | Male | 54 | No | Asymptomatic | Unvaccinated | / | / | / | / | 13 | 17.2 | 125.89 | 365.17 | 168.66 | 202.30 |
| BA.1 | Male | 11 | Yes | Asymptomatic | Unvaccinated | / | / | / | / | 7 | 35.55 | 10.00 | 10.00 | 21.13 | 10.00 |
| BA.1 | Female | 7 | No | Asymptomatic | Unvaccinated | / | / | / | / | 24 | 37.02 | 10.00 | 10.00 | 10.00 | 10.00 |
| BA.1 | Male | 4 | No | Asymptomatic | Unvaccinated | / | / | / | / | 11 | 38.23 | 10.00 | 516.29 | 10.00 | 10.00 |
| BA.1 | Male | 11 | No | Mild | Unvaccinated | / | / | / | / | 12 | 36.69 | 10.00 | 721.03 | 30.90 | 10.00 |
| BA.1 | Male | 10 | No | Mild | Unvaccinated | / | / | / | / | 27 | 18.78 | 10.00 | 114.55 | 42.17 | 10.00 |
| BA.1 | Female | 10 | No | Mild | Unvaccinated | / | / | / | / | 18 | 37.55 | 10.00 | 10.00 | 10.00 | 10.00 |
| BA.1 | Female | 47 | No | Asymptomatic | Regular | Inactivated | Inactivated | / | 295 | 11 | 37.3 | 870.96 | 1148.15 | 616.59 | 288.40 |
| BA.1 | Male | 14 | No | Asymptomatic | Regular | mRNA | mRNA | / | 180 | 8 | 22.4 | 186.16 | 53.70 | 21.38 | 20.00 |
| BA.1 | Female | 14 | No | Mild | Regular | Inactivated | Inactivated | / | 21 | 12 | 35.81 | 371.54 | 234.42 | 52.48 | 25.00 |
| BA.1 | Female | 12 | No | Asymptomatic | Regular | Inactivated | Inactivated | / | 12 | 11 | 34.32 | 43.65 | 269.15 | 57.54 | 10.00 |
| BA.1 | Male | 15 | No | Mild | Regular | mRNA | mRNA | / | 233 | 9 | 13.73 | 851.14 | 1047.13 | 446.68 | 309.00 |
| BA.1 | Female | 26 | No | Mild | Regular | Inactivated | Inactivated | / | NA | 8 | 15.35 | 173.78 | 1096.47 | 85.57 | 38.64 |
| BA.1 | Male | 36 | No | Mild | Regular | Inactivated | Inactivated | / | 225 | 7 | 14.18 | 54.95 | 812.80 | 19.45 | 10.00 |
| BA.1 | Male | 22 | No | Mild | Regular | Inactivated | Inactivated | / | 253 | 8 | 21.13 | 451.50 | 537.03 | 141.30 | 53.70 |
| BA.1 | Male | 23 | No | Mild | Regular | Inactivated | Inactivated | / | 253 | 7 | 15.43 | 10.00 | 10.00 | 10.00 | 10.00 |
| BA.1 | Female | 37 | No | Moderate | Regular | Inactivated | Inactivated | / | NA | 7 | 14.48 | 10.00 | 10.00 | 10.00 | 10.00 |
| BA.1 | Male | 39 | No | Moderate | Regular | Inactivated | Inactivated | / | 140 | 7 | 14.29 | 74.11 | 132.22 | 262.42 | 26.30 |
| BA.1 | Male | 49 | No | Moderate | Regular | Inactivated | Inactivated | / | NA | 7 | 16.87 | 10.00 | 10.00 | 10.00 | 250.60 |
| BA.1 | Male | 48 | No | Moderate | Regular | Inactivated | Inactivated | / | 204 | 8 | 28.65 | 52.97 | 126.77 | 93.11 | 64.60 |
| BA.1 | Male | 21 | No | Mild | Regular | mRNA | mRNA | / | 61 | 17 | 17.1 | 515.20 | 774.80 | 337.90 | 128.80 |
| BA.1 | Male | 28 | No | Moderate | Regular | mRNA | mRNA | / | 302 | 19 | 17.58 | 144.54 | 512.86 | 194.98 | 190.50 |
| BA.1 | Female | 53 | Yes | Moderate | Regular | mRNA | mRNA | / | 145 | 16 | 17.27 | 887.16 | 1234.24 | 810.59 | 204.20 |
| BA.1 | Female | 29 | No | Moderate | Regular | Inactivated | Inactivated | / | 198 | 7 | 21.39 | 478.63 | 575.44 | 37.15 | 66.10 |
| BA.1 | Male | 58 | Yes | Moderate | Regular | Inactivated | Inactivated | / | 210 | 14 | 19.49 | 208.93 | 194.98 | 43.65 | 63.10 |
| BA.1 | Male | 23 | No | Mild | Regular | mRNA | mRNA | / | 229 | 10 | 20.79 | 1513.56 | 5370.30 | 1862.10 | 1348.90 |
| BA.1 | Female | 28 | No | Asymptomatic | Regular | mRNA | mRNA | / | 233 | 27 | 16.15 | 1621.81 | 2238.72 | 2089.29 | 537.00 |
| BA.1 | Female | 47 | No | Moderate | Regular | mRNA | mRNA | / | 28 | 7 | 13.04 | 512.86 | 728.62 | 75.85 | 34.70 |
| BA.1 | Male | 23 | No | Mild | Regular | mRNA | mRNA | / | 147 | 26 | 14.85 | 630.95 | 810.59 | 371.54 | 172.20 |
| BA.1 | Male | 24 | No | Mild | Regular | mRNA | mRNA | / | NA | 27 | 17.77 | 1133.44 | 758.58 | 454.36 | 467.70 |
| BA.1 | Male | 59 | No | Moderate | Regular | Inactivated | Inactivated | / | 93 | 17 | 15.65 | 630.96 | 1273.50 | 1380.38 | 239.90 |
| BA.1 | Male | 72 | No | Moderate | Booster | Inactivated | Inactivated | Inactivated | 36 | 13 | 13.61 | 575.44 | 363.08 | 181.97 | 144.50 |
| BA.1 | Female | 20 | Yes | Moderate | Booster | Inactivated | Inactivated | Inactivated | 33 | 32 | 18.35 | 251.19 | 114.82 | 33.88 | 10.00 |
| BA.1 | Male | 46 | No | Moderate | Booster | Inactivated | Inactivated | Inactivated | 128 | 37 | 18.02 | 123.03 | 186.21 | 120.23 | 10.00 |
| BA.1 | Female | 47 | No | Mild | Booster | Inactivated | Inactivated | Inactivated | NA | 41 | 16.81 | 186.21 | 2454.71 | 107.89 | 10.00 |
| BA.1 | Male | 44 | Yes | Moderate | Booster | Inactivated | Inactivated | Inactivated | 10 | 8 | 16.9 | 275.42 | 794.00 | 158.49 | 36.30 |
| BA.1 | Male | 33 | No | Mild | Booster | Inactivated | Inactivated | mRNA | 53 | 16 | 14.66 | 407.38 | 1778.28 | 269.15 | 75.90 |
| BA.1 | Male | 22 | No | Moderate | Booster | Inactivated | Inactivated | mRNA | NA | 8 | 38.08 | 1804.00 | 1190.00 | 292.40 | 10.00 |
| BA.1 | Male | 44 | No | Moderate | Booster | Inactivated | Inactivated | Inactivated | 57 | 7 | 17.91 | 88.92 | 37.15 | 83.18 | 27.10 |
| BA.1 | Male | 34 | No | Mild | Booster | Inactivated | Inactivated | Inactivated | 310 | 12 | 15.78 | 181.97 | 131.83 | 250.61 | 10.00 |
| BA.1 | Male | 29 | No | Mild | Booster | Inactivated | Inactivated | mRNA | 43 | 25 | 16.51 | 1023.29 | 3194.48 | 1584.89 | 1621.80 |
| BA.1 | Male | 23 | No | Moderate | Booster | Inactivated | Inactivated | Inactivated | 189 | 14 | 14.17 | 1071.52 | 4786.30 | 794.33 | 1258.90 |
| BA.1 | Female | 48 | No | Moderate | Booster | mRNA | mRNA | mRNA | 26 | 7 | 15.35 | 3676.50 | 6075.50 | 2949.50 | 3548.10 |
| BA.1 | Female | 22 | No | Mild | Booster | Inactivated | Inactivated | mRNA | 47 | 20 | 15.82 | 30.13 | 295.12 | 77.45 | 128.80 |
| BA.1 | Female | 20 | No | Mild | Booster | Inactivated | Inactivated | mRNA | 47 | 20 | 16.57 | 622.44 | 774.46 | 239.88 | 173.80 |
| BA.2 | Female | 84 | Yes | Mild | Unvaccinated | / | / | / | / | 8 | 14.82 | 20.00 | 10.00 | 29.24 | 10.00 |
| BA.2 | Female | 34 | Yes | Asymptomatic | Unvaccinated | / | / | / | / | 8 | 34.76 | 23.51 | 10.00 | 10.00 | 30.90 |
| BA.2 | Male | 34 | No | Mild | Unvaccinated | / | / | / | / | 7 | 13.25 | 10.00 | 20.95 | 10.00 | 10.00 |
| BA.2 | Female | 31 | No | Moderate | Unvaccinated | / | / | / | / | 9 | 15.13 | 122.74 | 117.49 | 35.48 | 10.00 |
| BA.2 | Male | 41 | No | Asymptomatic | Unvaccinated | / | / | / | / | 9 | 23 | 52.48 | 32.55 | 645.65 | 562.30 |
| BA.2 | Male | 29 | No | Mild | Unvaccinated | / | / | / | / | 9 | 18.52 | 10.00 | 27.10 | 10.00 | 10.00 |
| BA.2 | Male | 60 | Yes | Mild | Unvaccinated | / | / | / | / | 10 | 17.3 | 10.00 | 10.00 | 10.00 | 10.00 |
| BA.2 | Female | 47 | Yes | Mild | Unvaccinated | / | / | / | / | 10 | 14.61 | 10.00 | 10.00 | 28.97 | 10.00 |
| BA.2 | Female | 50 | No | Moderate | Unvaccinated | / | / | / | / | 10 | 16.38 | 10.00 | 10.00 | 20.42 | 10.00 |
| BA.2 | Male | 48 | Yes | Moderate | Unvaccinated | / | / | / | / | 9 | 20.39 | 10.00 | 10.00 | 10.00 | 10.00 |
| BA.2 | Female | 37 | Yes | Mild | Unvaccinated | / | / | / | / | 11 | 16.79 | 10.00 | 10.00 | 10.00 | 10.00 |
| BA.2 | Male | 37 | No | Asymptomatic | Unvaccinated | / | / | / | / | 12 | 13.12 | 10.00 | 10.00 | 10.00 | 10.00 |
| BA.2 | Male | 64 | No | Moderate | Unvaccinated | / | / | / | / | 12 | 13.91 | 27.66 | 72.44 | 66.07 | 10.00 |
| BA.2 | Male | 24 | No | Mild | Unvaccinated | / | / | / | / | 13 | 18.2 | 21.95 | 57.20 | 56.79 | 43.70 |
| BA.2 | Female | 74 | Yes | Moderate | Unvaccinated | / | / | / | / | 13 | 16.68 | 10.00 | 10.00 | 10.00 | 20.00 |
| BA.2 | Female | 30 | No | Mild | Unvaccinated | / | / | / | / | 13 | 21.99 | 45.71 | 23.75 | 67.98 | 245.50 |
| BA.2 | Female | 16 | Yes | Mild | Unvaccinated | / | / | / | / | 13 | 13.01 | 10.00 | 10.00 | 10.00 | 10.00 |
| BA.2 | Male | 55 | No | Mild | Unvaccinated | / | / | / | / | 13 | 15.36 | 22.39 | 10.00 | 10.00 | 10.00 |
| BA.2 | Male | 34 | No | Mild | Unvaccinated | / | / | / | / | 12 | 20.11 | 21.38 | 54.95 | 91.20 | 51.90 |
| BA.2 | Male | 36 | No | Mild | Unvaccinated | / | / | / | / | 14 | 17.67 | 10.00 | 10.00 | 25.50 | 10.00 |
| BA.2 | Female | 52 | Yes | Asymptomatic | Unvaccinated | / | / | / | / | 14 | 12.39 | 20.95 | 33.88 | 26.30 | 20.00 |
| BA.2 | Female | 81 | Yes | Mild | Unvaccinated | / | / | / | / | 11 | 15.57 | 10.00 | 10.00 | 20.42 | 10.00 |
| BA.2 | Female | 68 | Yes | Mild | Unvaccinated | / | / | / | / | 15 | 16.98 | 38.02 | 75.86 | 112.20 | 10.00 |
| BA.2 | Female | 31 | No | Mild | Unvaccinated | / | / | / | / | 16 | 18.16 | 20.09 | 48.53 | 22.46 | 10.00 |
| BA.2 | Male | 39 | No | Asymptomatic | Unvaccinated | / | / | / | / | 16 | 16.05 | 10.00 | 10.00 | 20.70 | 55.00 |
| BA.2 | Male | 28 | No | Mild | Unvaccinated | / | / | / | / | 15 | 16.21 | 10.00 | 10.00 | 10.00 | 20.00 |
| BA.2 | Male | 56 | Yes | Asymptomatic | Unvaccinated | / | / | / | / | 15 | 17.84 | 22.39 | 232.92 | 192.31 | 457.10 |
| BA.2 | Male | 34 | No | Mild | Unvaccinated | / | / | / | / | 15 | 18.15 | 32.89 | 168.66 | 273.40 | 60.30 |
| BA.2 | Female | 50 | Yes | Mild | Unvaccinated | / | / | / | / | 15 | 16.49 | 32.36 | 64.28 | 43.91 | 37.20 |
| BA.2 | Male | 13 | No | Asymptomatic | Unvaccinated | / | / | / | / | 16 | 18.07 | 10.00 | 27.48 | 40.46 | 24.00 |
| BA.2 | Female | 32 | Yes | Mild | Unvaccinated | / | / | / | / | 17 | 14.56 | 315.14 | 194.98 | 161.22 | 26.30 |
| BA.2 | Male | 73 | Yes | Asymptomatic | Unvaccinated | / | / | / | / | 17 | 18.75 | 25.12 | 21.88 | 118.23 | 158.50 |
| BA.2 | Female | 41 | No | Mild | Unvaccinated | / | / | / | / | 17 | 17.19 | 10.00 | 10.00 | 22.24 | 10.00 |
| BA.2 | Female | 56 | Yes | Mild | Unvaccinated | / | / | / | / | 18 | 15.2 | 10.00 | 161.21 | 157.94 | 45.70 |
| BA.2 | Female | 91 | Yes | Mild | Unvaccinated | / | / | / | / | 23 | 27.06 | 21.13 | 194.98 | 141.25 | 74.10 |
| BA.2 | Female | 44 | No | Mild | Unvaccinated | / | / | / | / | 20 | 14.87 | 10.00 | 30.00 | 21.25 | 39.80 |
| BA.2 | Male | 5 | No | Mild | Unvaccinated | / | / | / | / | 18 | 19.83 | 10.00 | 44.67 | 53.70 | 21.00 |
| BA.2 | Male | 29 | No | Mild | Unvaccinated | / | / | / | / | 24 | 19.42 | 55.48 | 23.51 | 108.47 | 56.20 |
| BA.2 | Male | 10 | No | Mild | Unvaccinated | / | / | / | / | 26 | 16.81 | 10.00 | 10.00 | 10.00 | 10.00 |
| BA.2 | Female | 10 | No | Mild | Unvaccinated | / | / | / | / | 26 | 16.05 | 10.00 | 10.00 | 10.00 | 10.00 |
| BA.2 | Male | 71 | Yes | Moderate | Unvaccinated | / | / | / | / | 8 | 14.63 | 10.00 | 10.00 | 10.00 | 10.00 |
| BA.2 | Male | 26 | No | Mild | Unvaccinated | / | / | / | / | 10 | 15.83 | 10.00 | 10.00 | 10.00 | 10.00 |
| BA.2 | Female | 92 | Yes | Asymptomatic | Unvaccinated | / | / | / | / | 16 | 13.62 | 10.00 | 23.63 | 26.97 | 30.20 |
| BA.2 | Female | 30 | No | Asymptomatic | Unvaccinated | / | / | / | / | 47 | 22.27 | 398.11 | 69.02 | 59.27 | 70.79 |
| BA.2 | Male | 41 | No | Asymptomatic | Unvaccinated | / | / | / | / | 77 | 17.73 | 184.88 | 239.88 | 38.29 | 32.38 |
| BA.2 | Male | 53 | No | Asymptomatic | Unvaccinated | / | / | / | / | 53 | 19.95 | 22.24 | 10.00 | 52.19 | 10.00 |
| BA.2 | Male | 77 | Yes | Mild | Regular | Inactivated | Inactivated | / | 96 | 11 | 38.12 | 309.03 | 316.22 | 223.87 | 363.10 |
| BA.2 | Female | 69 | No | Mild | Regular | Inactivated | Inactivated | / | 21 | 10 | 36.6 | 52.48 | 67.61 | 140.28 | 20.00 |
| BA.2 | Male | 11 | No | Asymptomatic | Regular | Inactivated | Inactivated | / | 22 | 13 | 38.02 | 10.00 | 57.54 | 89.13 | 37.20 |
| BA.2 | Male | 14 | No | Asymptomatic | Regular | mRNA | mRNA | / | 28 | 8 | 35.67 | 1845.05 | 831.76 | 1071.52 | 724.40 |
| BA.2 | Female | 68 | Yes | Mild | Regular | Inactivated | Inactivated | / | 177 | 8 | 15.16 | 107.15 | 62.95 | 56.23 | 26.70 |
| BA.2 | Female | 14 | No | Mild | Regular | Inactivated | Inactivated | / | NA | 9 | 14.62 | 81.28 | 61.66 | 125.89 | 81.30 |
| BA.2 | Male | 13 | No | Mild | Regular | Inactivated | Inactivated | / | 175 | 9 | 15.16 | 1513.56 | 562.34 | 954.99 | 1148.20 |
| BA.2 | Male | 14 | No | Mild | Regular | Inactivated | Inactivated | / | 187 | 10 | 12.19 | 30.81 | 123.03 | 75.63 | 100.00 |
| BA.2 | Male | 70 | No | Mild | Regular | Inactivated | Inactivated | / | 102 | 14 | 13.62 | 316.23 | 245.47 | 234.42 | 645.70 |
| BA.2 | Male | 67 | No | Asymptomatic | Regular | Inactivated | Inactivated | / | 198 | 34 | 18.02 | 676.08 | 446.68 | 812.83 | 549.50 |
| BA.2 | Male | 9 | No | Mild | Regular | Inactivated | Inactivated | / | 68 | 10 | 25.22 | 35.48 | 87.09 | 107.16 | 43.70 |
| BA.2 | Female | 7 | No | Mild | Regular | Inactivated | Inactivated | / | 108 | 80 | 15.63 | 22.46 | 24.12 | 29.99 | 26.30 |
| BA.2 | Female | 9 | No | Asymptomatic | Regular | Inactivated | Inactivated | / | 49 | 78 | 20.8 | 82.87 | 165.96 | 177.83 | 56.89 |
| BA.2 | Female | 18 | No | Asymptomatic | Regular | mRNA | mRNA | / | 293 | 11 | 38.91 | 345.30 | 363.33 | 374.54 | 273.40 |
| BA.2 | Male | 9 | No | Mild | Regular | Inactivated | Inactivated | / | 121 | 85 | 14.67 | 515.82 | 313.55 | 161.21 | 117.65 |
| BA.2 | Female | 8 | No | Asymptomatic | Regular | Inactivated | Inactivated | / | 92 | 91 | 20.84 | 40.91 | 83.18 | 100.51 | 42.17 |
| BA.2 | Male | 59 | No | Mild | Regular | Inactivated | Inactivated | / | 205 | 62 | 16.15 | 10.00 | 224.23 | 101.02 | 136.95 |
| BA.2 | Male | 8 | No | Mild | Regular | Inactivated | Inactivated | / | 38 | 87 | 23.83 | 29.11 | 58.88 | 64.94 | 104.69 |
| BA.2 | Male | 6 | No | Mild | Regular | Inactivated | Inactivated | / | 112 | 75 | 17.8 | 58.08 | 50.89 | 105.22 | 62.99 |
| BA.2 | Female | 30 | No | Asymptomatic | Regular | Inactivated | Inactivated | / | 268 | 79 | 24.65 | 164.51 | 128.26 | 62.03 | 44.15 |
| BA.2 | Female | 18 | No | Mild | Regular | Inactivated | Inactivated | / | NA | 59 | 14.49 | 473.15 | 588.71 | 214.24 | 130.89 |
| BA.2 | Female | 34 | No | Mild | Regular | Inactivated | Inactivated | / | 270 | 89 | 16.26 | 2990.20 | 973.42 | 556.67 | 233.56 |
| BA.2 | Female | 14 | No | Mild | Regular | Inactivated | Inactivated | / | 194 | 72 | 22.46 | 343.40 | 176.64 | 319.96 | 273.40 |
| BA.2 | Male | 5 | No | Asymptomatic | Regular | Inactivated | Inactivated | / | 96 | 75 | 23.94 | 173.78 | 56.61 | 269.22 | 109.57 |
| BA.2 | Male | 10 | No | Mild | Regular | Inactivated | Inactivated | / | 111 | 88 | 18.53 | 163.68 | 3627.44 | 363.33 | 171.32 |
| BA.2 | Female | 29 | No | Asymptomatic | Regular | Inactivated | Inactivated | / | 144 | 47 | 14.05 | 97.72 | 10.00 | 179.35 | 36.39 |
| BA.2 | Male | 5 | No | Asymptomatic | Regular | Inactivated | Inactivated | / | 119 | 86 | 24.54 | 194.98 | 139.83 | 104.71 | 71.52 |
| BA.2 | Female | 24 | No | Mild | Regular | Inactivated | Inactivated | / | 388 | 9 | 28.04 | 579.43 | 2960.06 | 861.59 | 218.63 |
| BA.2 | Male | 54 | No | Mild | Regular | mRNA | mRNA | / | 301 | 8 | 17.33 | 613.06 | 5308.84 | 1082.93 | 1491.08 |
| BA.2 | Male | 8 | No | Asymptomatic | Regular | Inactivated | Inactivated | / | 227 | 7 | 20.87 | 776.25 | 576.77 | 724.94 | 619.30 |
| BA.2 | Male | 48 | No | Asymptomatic | Regular | Inactivated | Inactivated | / | 332 | 9 | 18.72 | 741.31 | 3036.69 | 1139.20 | 380.28 |
| BA.2 | Male | 31 | No | Asymptomatic | Regular | Inactivated | Inactivated | / | 297 | 37 | 15.2 | 273.40 | 570.95 | 336.67 | 50.37 |
| BA.2 | Male | 32 | No | Asymptomatic | Regular | Protein subunit | Protein subunit | Protein subunit | 278 | 37 | 19.04 | 1659.59 | 1560.99 | 2594.18 | 1353.94 |
| BA.2 | Male | 47 | No | Asymptomatic | Regular | Protein subunit | Protein subunit | Protein subunit | 313 | 37 | 15.48 | 370.77 | 1288.25 | 341.19 | 91.20 |
| BA.2 | Female | 34 | No | Mild | Booster | Inactivated | Inactivated | Inactivated | 77 | 87 | 17.06 | 273.40 | 570.95 | 336.67 | 50.37 |
| BA.2 | Male | 5 | No | Mild | Booster | Inactivated | Inactivated | Inactivated | 26 | 82 | 20.32 | 161.21 | 141.97 | 198.52 | 76.40 |
| BA.2 | Female | 40 | No | Asymptomatic | Booster | Inactivated | Inactivated | Inactivated | 37 | 8 | 20.02 | 220.80 | 104.69 | 109.57 | 80.37 |
| BA.2 | Male | 47 | No | Mild | Booster | Inactivated | Inactivated | Inactivated | 230 | 9 | 14.44 | 346.74 | 59.87 | 73.35 | 137.28 |
| BA.2 | Male | 51 | No | Asymptomatic | Booster | Inactivated | Inactivated | Inactivated | 249 | 12 | 17.81 | 128.82 | 20.00 | 67.28 | 59.95 |
| BA.2 | Male | 22 | No | Mild | Booster | mRNA | mRNA | mRNA | 136 | 8 | 15.7 | 5700.33 | 1931.97 | 1360.82 | 591.70 |
| BA.2 | Male | 33 | No | Mild | Booster | mRNA | mRNA | mRNA | 196 | 7 | 22.19 | 402.16 | 418.89 | 582.64 | 198.52 |
| BA.2 | Female | 39 | No | Mild | Booster | Inactivated | Inactivated | Inactivated | 202 | 8 | 16.28 | 736.04 | 1513.91 | 699.68 | 380.28 |
| BA.2 | Female | 50 | No | Asymptomatic | Booster | Inactivated | Inactivated | Inactivated | 136 | 10 | 16.06 | 326.59 | 857.24 | 883.69 | 1403.14 |
| BA.2 | Male | 38 | No | Asymptomatic | Booster | Inactivated | Inactivated | Inactivated | 218 | 10 | 20.41 | 237.14 | 2391.66 | 831.57 | 1055.60 |
| BA.2 | Female | 19 | No | Asymptomatic | Booster | Inactivated | Inactivated | Inactivated | 182 | 7 | 17.15 | 338.38 | 1855.24 | 1818.03 | 1468.59 |
| BA.2 | Male | 25 | No | Mild | Booster | Inactivated | Inactivated | Inactivated | 85 | 9 | 16.35 | 531.74 | 794.33 | 374.54 | 396.10 |
| BA.2 | Male | 22 | No | Mild | Booster | Inactivated | Inactivated | Inactivated | 132 | 9 | 18.46 | 467.74 | 778.39 | 794.33 | 1976.97 |
| BA.2 | Male | 35 | No | Mild | Booster | Inactivated | Inactivated | Inactivated | 35 | 34 | 17.96 | 427.46 | 524.81 | 446.68 | 208.93 |
| BA.2 | Male | 33 | No | Mild | Booster | Inactivated | Inactivated | Inactivated | 104 | 37 | 18.46 | 101.53 | 24.55 | 194.98 | 163.68 |
| BA.2 | Male | 49 | No | Mild | Booster | Inactivated | Inactivated | Inactivated | 25 | 34 | 17.71 | 939.29 | 1949.84 | 269.15 | 72.44 |
| BA.2 | Female | 32 | No | Asymptomatic | Booster | Inactivated | Inactivated | Inactivated | 181 | 75 | 15.2 | 49.61 | 77.62 | 43.65 | 10.00 |
| BA.2 | Male | 26 | No | Mild | Booster | Inactivated | Inactivated | Inactivated | 84 | 37 | 20.26 | 96.52 | 131.83 | 42.66 | 110.66 |
| BA.2 | Female | 42 | No | Mild | Booster | Inactivated | Inactivated | Inactivated | 84 | 91 | 22.1 | 45.05 | 10.00 | 75.09 | 61.72 |
| BA.2 | Female | 33 | No | Asymptomatic | Booster | Inactivated | Inactivated | Inactivated | 85 | 72 | 15.14 | 328.40 | 786.32 | 179.35 | 102.33 |
| BA.2 | Female | 38 | No | Mild | Booster | Inactivated | Inactivated | Inactivated | 139 | 78 | 17.6 | 116.47 | 120.06 | 126.33 | 24.37 |
| BA.2 | Male | 51 | No | Mild | Booster | Inactivated | Inactivated | Inactivated | 184 | 82 | 18.26 | 37.33 | 214.24 | 91.26 | 42.38 |
| BA.2 | Male | 56 | Yes | Mild | Booster | Inactivated | Inactivated | Inactivated | 91 | 56 | 16.28 | 88.98 | 402.16 | 182.10 | 44.37 |
| BA.2 | Male | 48 | No | Mild | Booster | Inactivated | Inactivated | Inactivated | 55 | 59 | 15.66 | 152.44 | 69.37 | 48.87 | 74.11 |
| BA.2 | Male | 34 | No | Asymptomatic | Booster | Inactivated | Inactivated | Inactivated | 17 | 37 | 17.73 | 169.59 | 141.97 | 42.60 | 25.63 |
| BA.2 | Male | 48 | No | Mild | Booster | Inactivated | Inactivated | Inactivated | 124 | 62 | 15.58 | 336.67 | 352.45 | 4377.24 | 250.78 |
| BA.2 | Male | 36 | No | Mild | Booster | Inactivated | Inactivated | Inactivated | 34 | 85 | 16.39 | 130.89 | 35.48 | 295.12 | 128.82 |
| BA.2 | Male | 42 | No | Mild | Booster | Inactivated | Inactivated | Inactivated | NA | 82 | 20.18 | 184.08 | 473.15 | 2715.19 | 824.14 |
| BA.2 | Male | 36 | No | Mild | Booster | Inactivated | Inactivated | Inactivated | 84 | 9 | 18.2 | 6025.59 | 407.38 | 1819.70 | 1023.30 |
| BA.2 | Male | 47 | No | Asymptomatic | Booster | Inactivated | Inactivated | Inactivated | 183 | 9 | 16.14 | 70.79 | 107.15 | 116.41 | 104.71 |
| BA.2 | Male | 57 | Yes | Asymptomatic | Booster | Inactivated | Inactivated | Inactivated | 116 | 10 | 14.76 | 912.01 | 103.99 | 280.54 | 512.90 |
| BA.2 | Female | 40 | No | Mild | Booster | Inactivated | Inactivated | Inactivated | 159 | 10 | 18.73 | 75.85 | 85.11 | 147.91 | 141.30 |
| BA.2 | Male | 72 | Yes | Mild | Booster | Inactivated | Inactivated | Inactivated | 123 | 12 | 33.98 | 2398.83 | 408.32 | 706.32 | 323.60 |
| BA.2 | Female | 42 | No | Asymptomatic | Booster | Inactivated | Inactivated | Inactivated | NA | 11 | 23.01 | 352.37 | 87.10 | 213.79 | 407.40 |
| BA.2 | Male | 56 | No | Mild | Booster | Inactivated | Inactivated | Inactivated | 99 | 11 | 14.16 | 301.99 | 110.66 | 263.00 | 181.10 |
| BA.2 | Female | 35 | No | Mild | Booster | Inactivated | Inactivated | Inactivated | 28 | 12 | 15.6 | 245.47 | 161.80 | 151.36 | 48.90 |
| BA.2 | Male | 45 | No | Mild | Booster | Inactivated | Inactivated | Inactivated | 106 | 12 | 17.7 | 239.88 | 70.79 | 44.56 | 125.00 |
| BA.2 | Female | 40 | No | Mild | Booster | Inactivated | Inactivated | Inactivated | 87 | 12 | 15.87 | 160.10 | 233.90 | 117.30 | 30.20 |
| BA.2 | Male | 37 | No | Mild | Booster | Inactivated | Inactivated | Inactivated | 59 | 13 | 12.85 | 346.73 | 74.13 | 407.38 | 501.20 |
| BA.2 | Male | 42 | No | Asymptomatic | Booster | Inactivated | Inactivated | Inactivated | 171 | 14 | 21.61 | 401.79 | 389.05 | 363.08 | 245.50 |
| BA.2 | Female | 27 | No | Asymptomatic | Booster | Inactivated | Inactivated | mRNA | 113 | 14 | 16.45 | 134.89 | 112.20 | 131.83 | 33.10 |
| BA.2 | Female | 41 | No | Mild | Booster | mRNA | mRNA | mRNA | 17 | 16 | 16.58 | 1148.15 | 741.31 | 1011.57 | 436.50 |

**NA: Not available.**
